# Female sex–related stroke risk in patients with atrial flutter—comparison between CHA_2_DS_2_-VA and CHA_2_DS_2_-VASc scores

**DOI:** 10.1093/europace/euag121

**Published:** 2026-05-19

**Authors:** Aapo Ounaslehto, Hermon Eyob Fesseha, Ville Langén, Aapo L Aro, Jussi Jaakkola, Eero Jalli, Olli Halminen, Birgitta Salmela, Rasmus Siponen, Jari Haukka, Jukka Putaala, Miika Linna, Pirjo Mustonen, Juha Hartikainen, K E Juhani Airaksinen, Mika Lehto, Konsta Teppo

**Affiliations:** Department of Internal Medicine, Turku University Hospital, Kiinamyllynkatu 10, Turku 20520, Finland; University of Helsinki, Helsinki, Finland; Division of Medicine, Turku University Hospital and University of Turku, Turku, Finland; Heart and Lung Center, Helsinki University Hospital and University of Helsinki, Helsinki, Finland; Cardiac Unit, Department of Internal Medicine, Satasairaala, Pori, Finland; Cardiac Unit, Department of Internal Medicine, Satasairaala, Pori, Finland; University of Turku, Turku, Finland; Department of Health and Social Management, Faculty of Social Sciences and Business Studies, University of Eastern Finland, Kuopio, Finland; Heart Center, Department of Internal Medicine, Päijät-Häme Central Hospital, Lahti, Finland; University of Helsinki, Helsinki, Finland; University of Helsinki, Helsinki, Finland; Neurology, Helsinki University Hospital and University of Helsinki, Helsinki, Finland; Department of Health and Social Management, Faculty of Social Sciences and Business Studies, University of Eastern Finland, Kuopio, Finland; Aalto University, Espoo, Finland; Turku University Hospital and University of Turku, Turku, Finland; Kuopio University Hospital and University of Eastern Finland, Helsinki, Finland; Turku University Hospital and University of Turku, Turku, Finland; Department of Internal Medicine, Jorvi Hospital, HUS Helsinki University Hospital and University of Helsinki, Helsinki, Finland; Cardiac Unit, Department of Internal Medicine, Satasairaala, Pori, Finland; Department of Internal Medicine, University of Turku, Turku, Finland

**Keywords:** Atrial flutter, Women, Ischaemic stroke, CHA2DS2-VA, CHA2DS2-VASc

Recent studies have shown that the stroke risk traditionally associated with female sex in patients with atrial fibrillation has decreased and is no longer evident in contemporary cohorts.^1–3^ Correspondingly, the sex-neutral CHA_2_DS_2_-VA score has been shown to perform comparably to the CHA_2_DS_2_-VASc score in predicting stroke risk in contemporary patients with atrial fibrillation.^3–5^ However, specifically in patients with atrial flutter (AFL), the role of female sex in stroke risk stratification is unclear. We conducted a cohort study to evaluate the association between female sex and stroke risk, assess temporal trends in this association, and compare the accuracy of the CHA_2_DS_2_-VA and CHA_2_DS_2_-VASc scores for stroke risk estimation in patients with electrocardiographically (ECG) confirmed AFL.

The Finnish AntiCoagulation in Atrial Fibrillation (FinACAF) is a nationwide registry-linkage retrospective cohort study covering all 229 565 patients with incident AF or AFL in Finland from 2007 to 2018.^6^ Within the incident cohort, digitized ECGs were available from the Helsinki and Uusimaa Hospital district from 52 583 patients. ECGs that were digitally interpreted as poor quality, paediatric ECGs, and ECGs presenting a junctional or unspecified rhythm were excluded. Thereafter, since the focus of the current study was on patients with only AFL, those with ECGs showing atrial fibrillation, only sinus rhythm, or only paced rhythms were subsequently excluded. Follow-up began at the first AFL diagnosis and continued until the first ischaemic stroke, death, or 31 December 2018, whichever occurred first. Additionally, when comparing the risk scores, we performed separate analyses restricted to follow-up without anticoagulant therapy. Data on baseline characteristics, anticoagulant use and stroke outcome were obtained from national registers, using the same definitions as in previous FinACAF studies.^1^ The study protocol was approved by the Ethics Committee of the Medical Faculty of Helsinki University, Helsinki, Finland (nr. 15/2017).

Stroke incidence was modelled using Poisson regression, with adjusted analyses including sex, age, calendar year, heart failure, hypertension, diabetes, prior ischaemic stroke or transient ischaemic attack, vascular disease, dyslipidaemia, prior bleeding, alcohol use disorder, renal failure, liver disease, cancer, dementia, psychiatric disorders, income level, and anticoagulant use. Interaction between calendar year period and sex was tested to assess whether the association between sex and stroke rate changed over time. The stroke risk scores were treated as categorical variables and compared using Harrell’s *c*-statistic from Cox regression, as well as categorical net reclassification index (NRI; annual risk thresholds of 1% and 2%), continuous NRI and integrated discrimination improvement (IDI). Event probabilities were derived from the Cox models. Analyses were performed in R version 4.0.5 (R Core Team, Vienna, Austria; https://www.R-project.org).

We identified 2409 patients with new-onset AFL (38.8% women; mean follow-up 3.4 years). Women were of similar age to men [68.1 (SD 12.5) vs. 68.9 (SD 12.7) years] but had a higher burden of comorbidities, reflected by higher CHA_2_DS_2_-VA scores [3.0 (SD 1.7) vs. 2.4 (SD 1.7)]. Initiation of anticoagulant therapy was similar between men (72.2%) and women (69.3%, *P* = 0.12). Overall, 88 patients (3.7%) experienced an ischaemic stroke (41 women and 47 men). Female sex was associated with a higher stroke rate in unadjusted analyses [IRR 1.57 (1.03–2.39)] but not after adjustment for confounders [adjusted IRR 1.21 (0.76–1.93); *Figure 1*]. A borderline statistically significant interaction between calendar year period (2007–2012 vs. 2013–2018) and sex was observed (*P* = 0.05), and female sex was independently associated with stroke risk in 2007–2012 but not in 2013–2018 (*Figure 1*).

**Figure 1:**
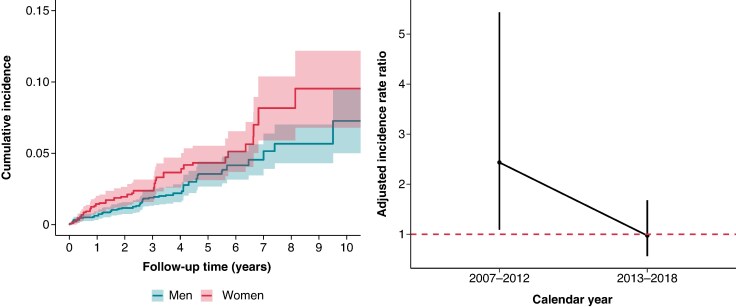
Incidence of ischaemic stroke in men and women with atrial flutter. The left panel shows the crude cumulative incidence of stroke in men and women. The right panel shows adjusted incidence rate ratios of ischaemic stroke comparing women with men across calendar year periods (red dashed reference line for men). Shaded areas and error bars represent 95% confidence intervals. Adjusted analyses included age, calendar year, heart failure, hypertension, diabetes, prior ischaemic stroke or transient ischaemic attack, vascular disease, dyslipidaemia, prior bleeding, alcohol use disorder, renal failure, liver disease, cancer, dementia, psychiatric disorders, income level, and anticoagulant use. Women are compared with men in the adjusted incidence rate ratios of the right panel.

When comparing CHA_2_DS_2_-VA and CHA_2_DS_2_-VASc scores over the entire follow-up, there were no significant differences in IDI [0.00 (−0.061–0.084)], continuous NRI [0.01 (−0.67–0.73)], or categorical NRI [−0.18 (−0.41–0.17)]. *C*-statistics were similar for both scores (0.63 vs. 0.63, *P* = 0.65), with consistent results when the study period was divided into 2007–2012 (0.66 vs. 0.68, *P* = 0.61) and 2013–2018 (0.64 vs. 0.65, *P* = 0.56). Correspondingly, during follow-up without anticoagulant therapy, no significant differences were observed between the scores for IDI [0.01 (−0.14–0.23)], continuous NRI [−0.34 (−1.36–1.33)], or categorical NRI [0.01 (−0.41–0.26)]. *C*-statistics were similar (CHA_2_DS_2_-VA 0.65 vs. CHA_2_DS_2_-VASc 0.66, *P* = 0.75), with consistent values in 2007–2012 (0.68 vs. 0.69, *P* = 0.56) and 2013–2018 (0.60 vs. 0.65, *P* = 0.42).

The findings of the current study in patients with AFL are consistent with the growing body of evidence in atrial fibrillation, indicating a decreasing role of female sex in stroke risk stratification.^1–3^ Moreover, as previously observed in atrial fibrillation, our study shows that the CHA_2_DS_2_-VA and CHA_2_DS_2_-VASc scores perform comparably in predicting stroke risk also specifically in patients with AFL.^3–5^

Notably, oral anticoagulation has historically been initiated less frequently in women than in men, and the inclusion of sex as a component of the CHA_2_DS_2_-VASc score may have contributed to increased anticoagulant use among women.^7,8^ In Finland, such sex-related disparities have been minimal and appear to have been eliminated over time.^8^

Strengths of our study include the use of a well-validated hospital care register to define stroke outcomes and the confirmation of AFL by ECG recordings rather than relying solely on diagnostic codes.^9^ Nevertheless, register-based studies have inherent limitations, including information bias from inaccuracies in the registry data. Relatedly, residual confounding from unmeasured factors, such as incident comorbidities or changes in pharmacotherapy during follow-up, cannot be excluded. Patients with AFL are often treated with catheter ablation and may later develop atrial fibrillation, which could influence stroke risk, although no ECG-verified atrial fibrillation occurred in this cohort.^10^ Data on AFL subtype were unavailable. Finally, the exclusive focus on AFL resulted in a relatively small sample size, potentially limiting statistical power. Larger studies in more diverse populations and registries are needed to confirm these findings.

In this retrospective cohort study, the previously observed excess stroke risk associated with female sex appears to have decreased and is not evident in more contemporary patients with AFL. Moreover, the CHA_2_DS_2_-VA and CHA_2_DS_2_-VASc scores perform similarly in predicting stroke. These findings suggest that the CHA_2_DS_2_-VA score may offer a simpler yet clinically comparable approach to stroke risk assessment and decision-making on oral anticoagulant therapy in patients with AFL.

## Data Availability

Because of the sensitive nature of the data collected for this study, requests to access the dataset from qualified researchers trained in human subject confidentiality protocols may be sent to the Finnish national register holders (KELA, Finnish Institute for Health and Welfare, Population Register Center and Tax Register) through Findata (https://findata.fi/en/).
